# Supplementary material for: AGS-v PLUS, a Mosquito Salivary Peptide Vaccine, Modulates the Response to Aedes Mosquito Bites in Humans
Source: Vaccines (Basel). 2025 Sep 30;13(10):1026. doi: 10.3390/vaccines13101026 (PMC12567680; doi:10.3390/vaccines13101026)

Figure S1- Mapping of DEGs to known biological pathways identifies functional modules in mosquito bitten skin compared to resting skin.

(A) Immune / inflammatory signaling pathways

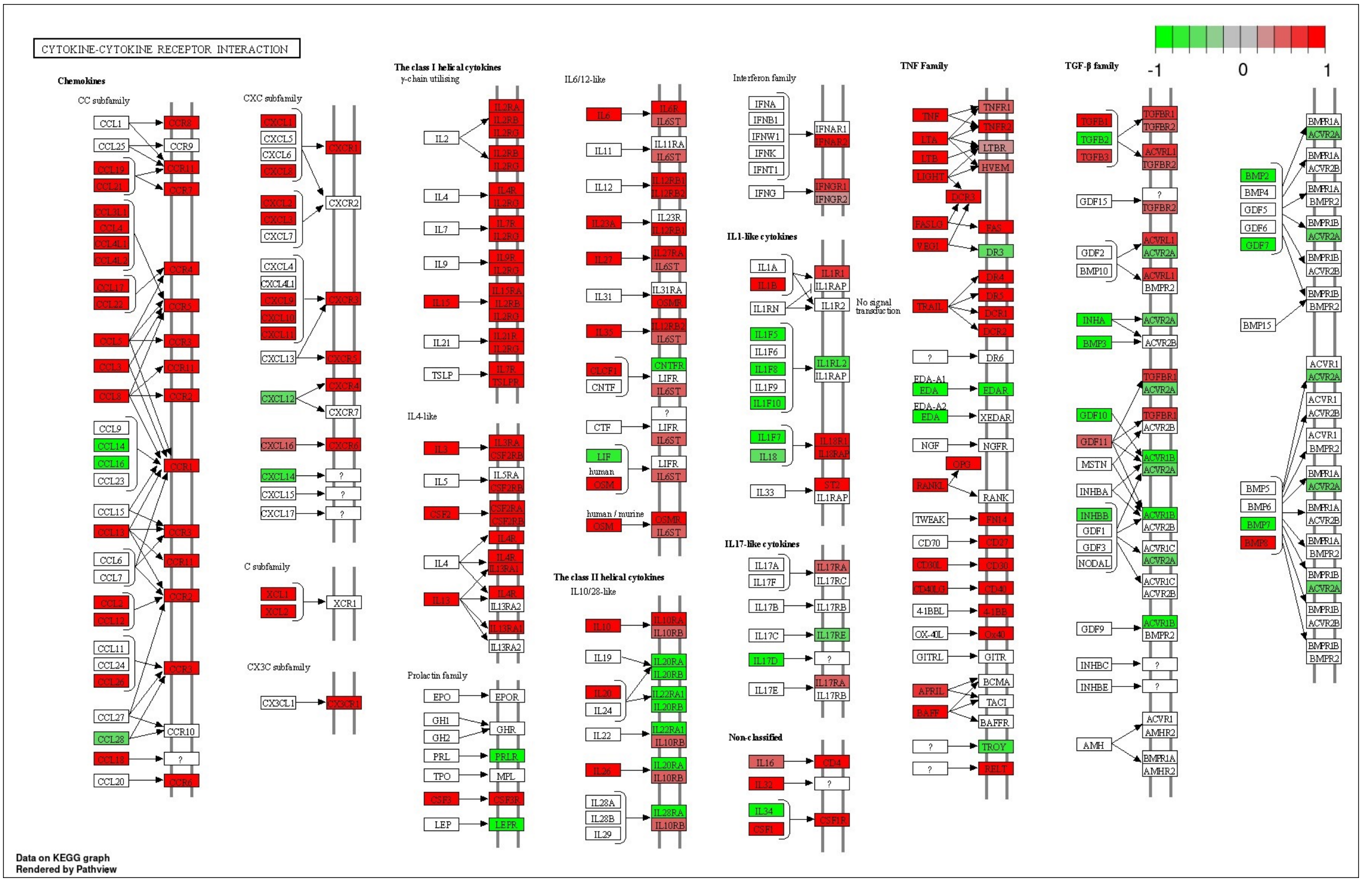

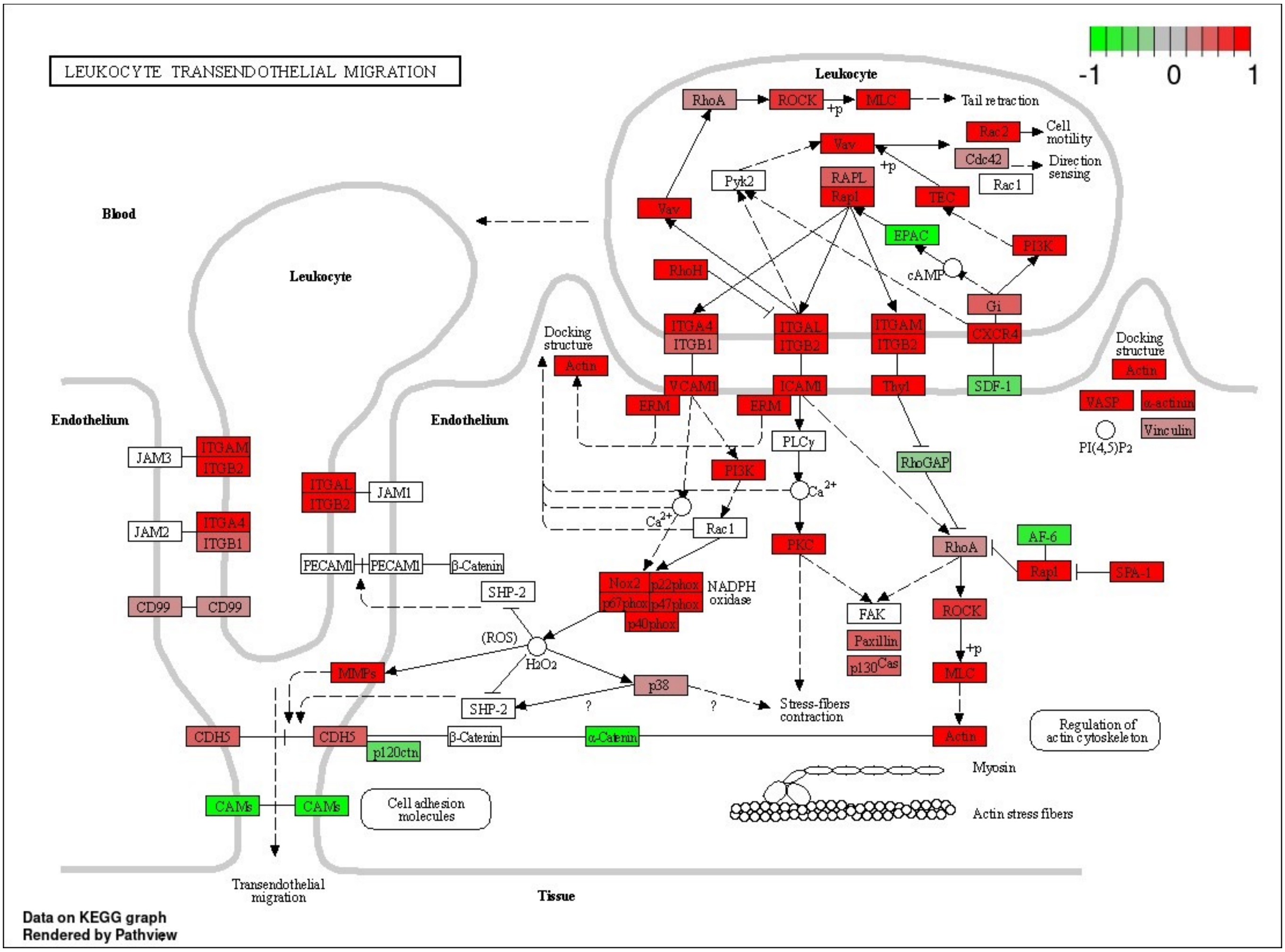

Figure S1A Immune / inflammatory signaling pathways (continued)

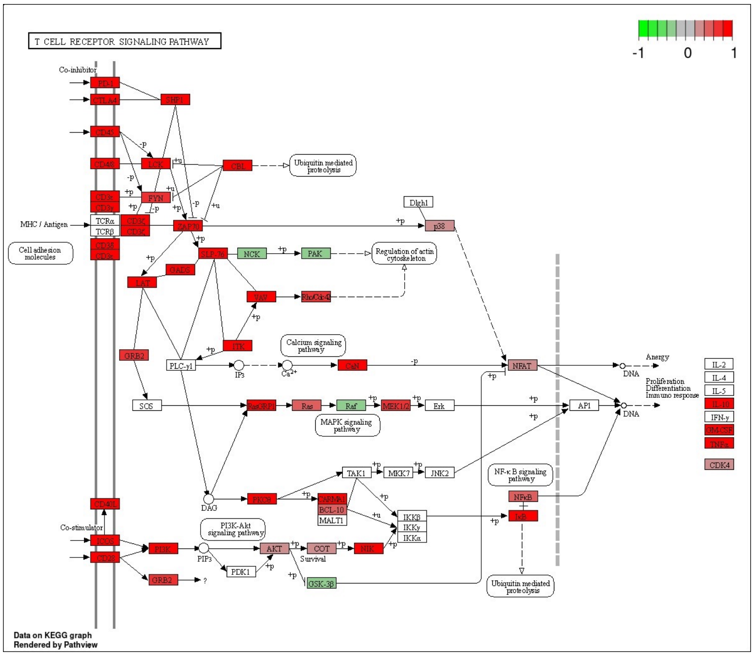

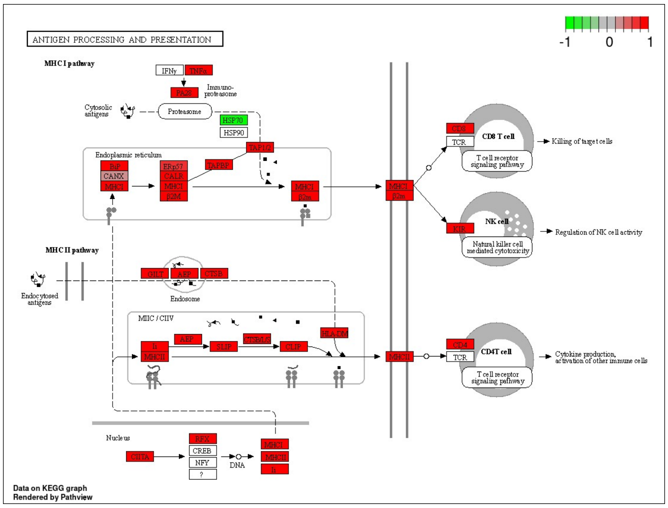

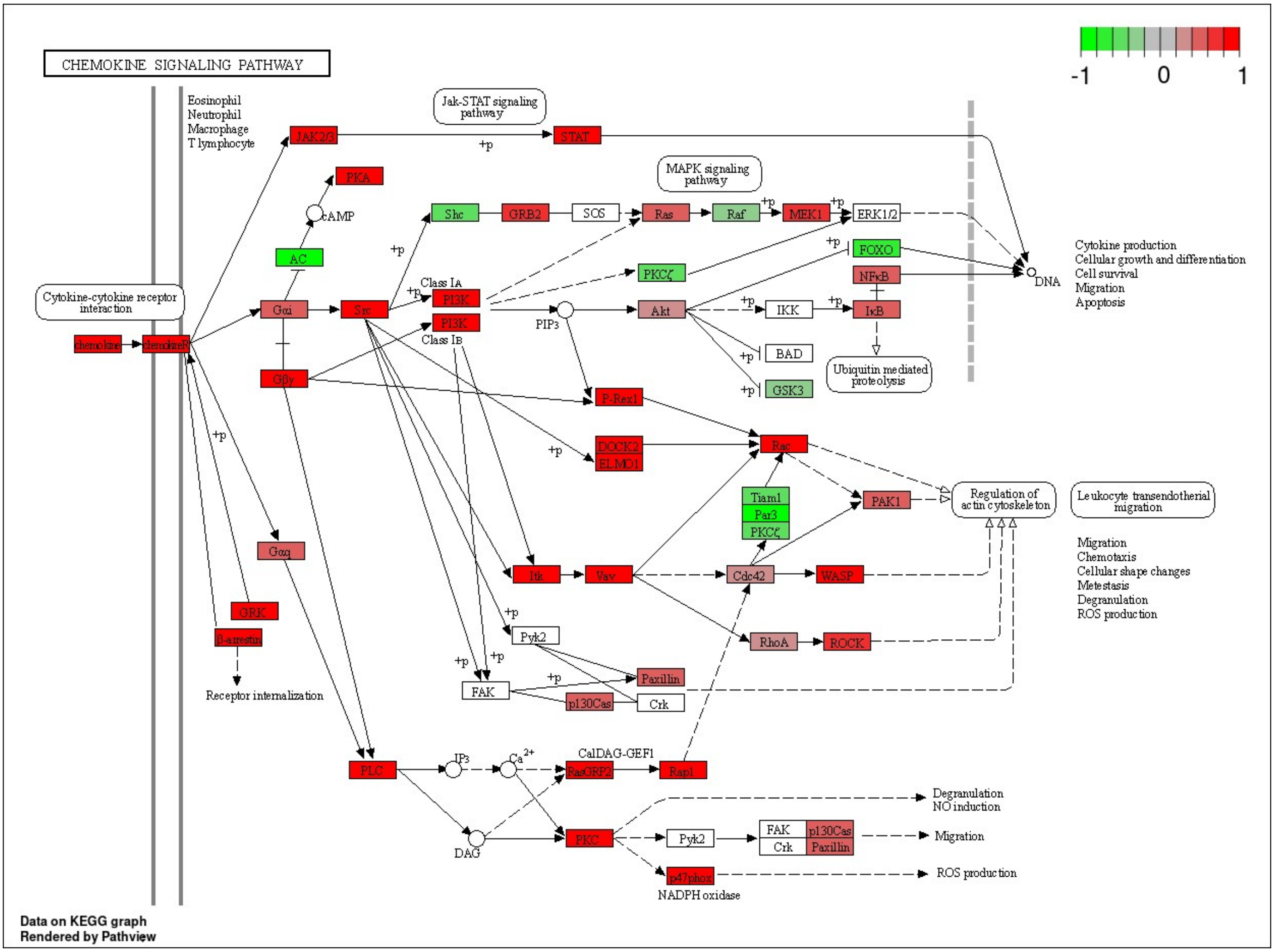

Figure S1A Immune / inflammatory signaling pathways (continued)

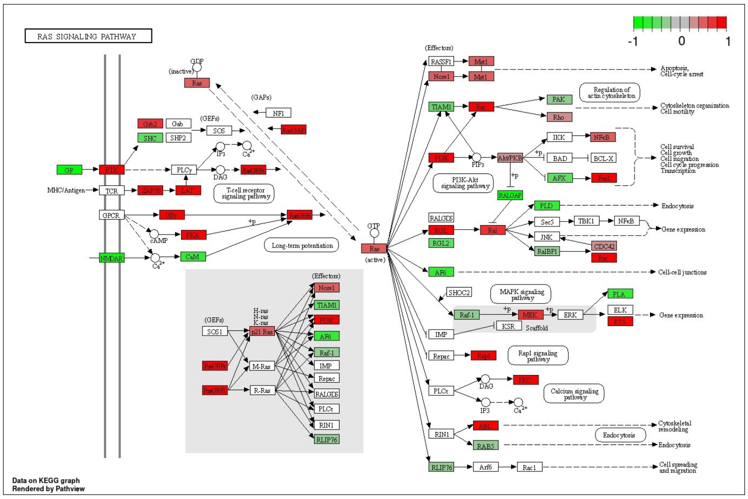

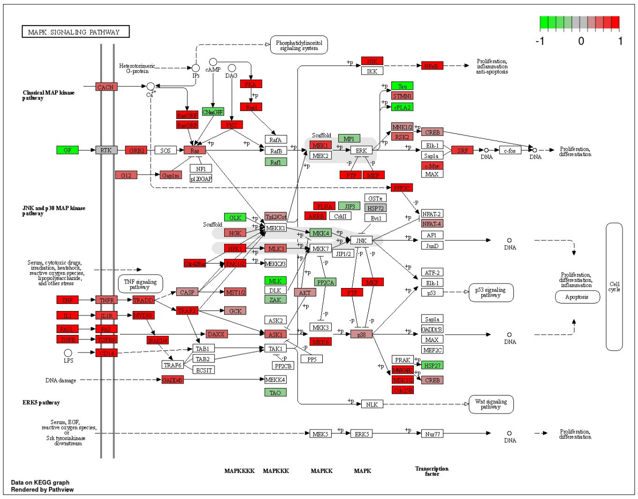

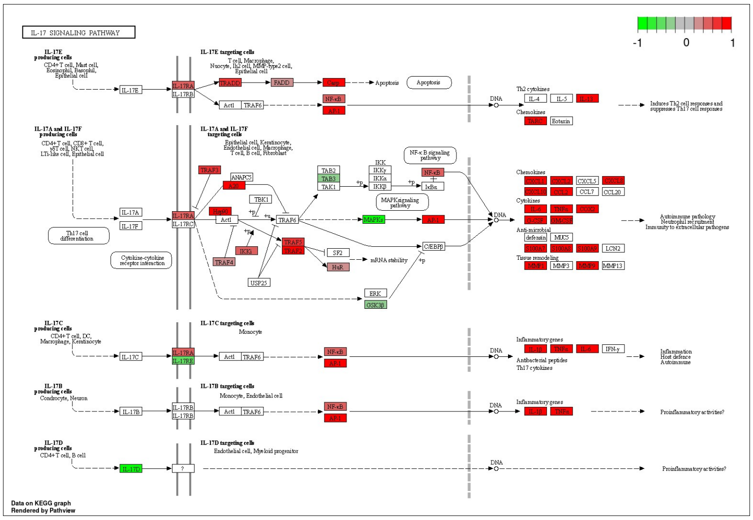

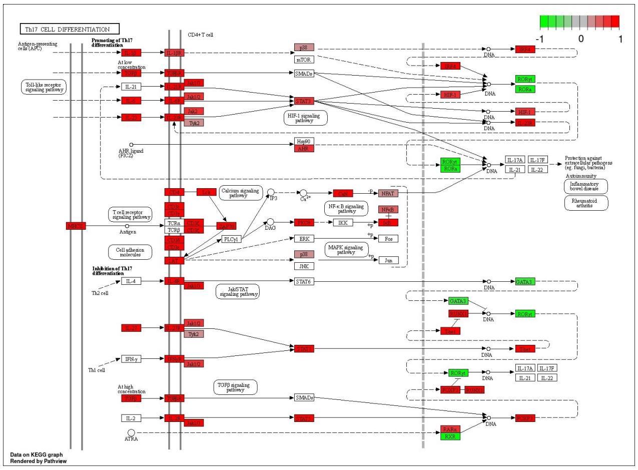

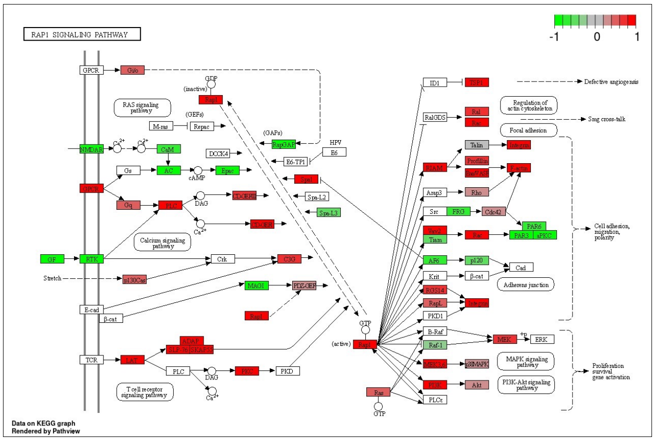

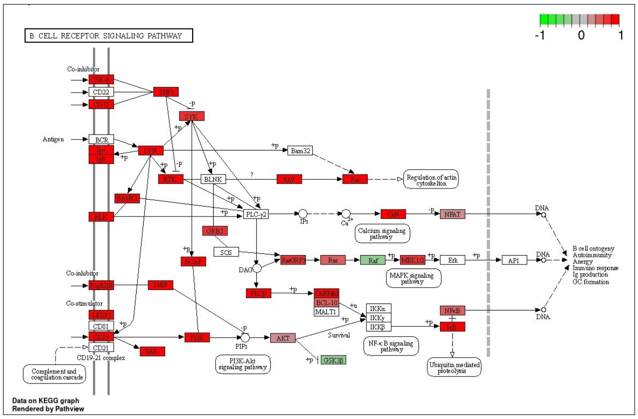

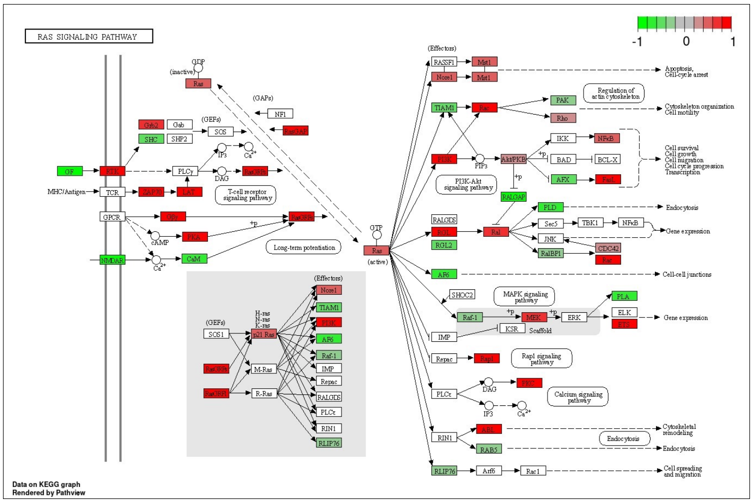

Figure S1A Immune / inflammatory signaling pathways (continued)

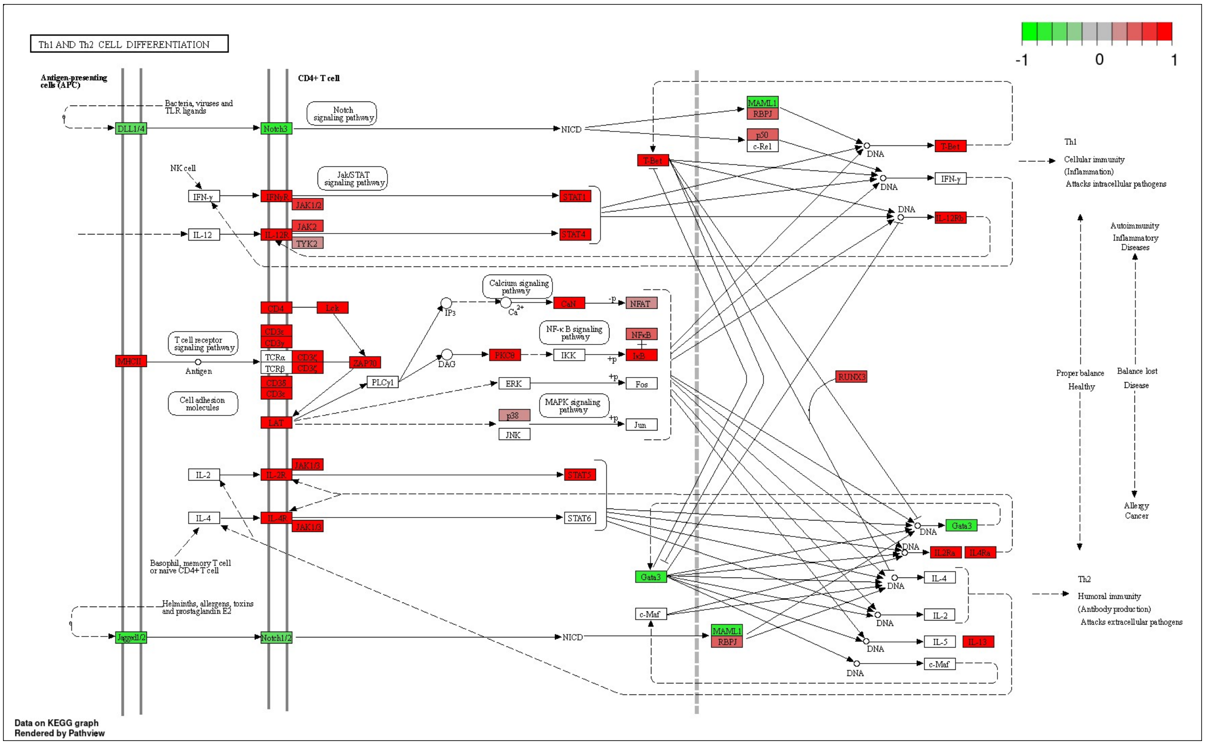

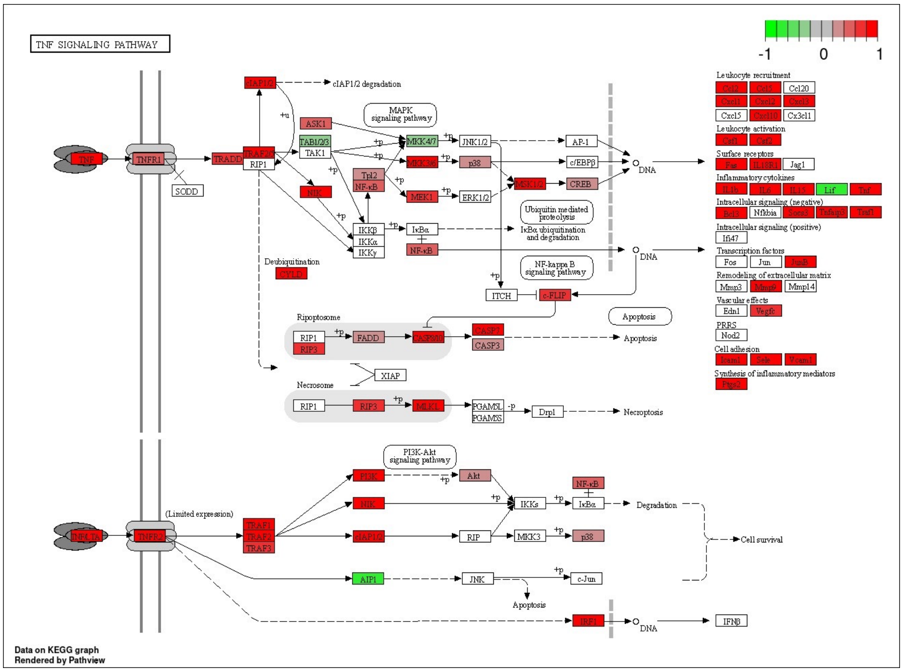

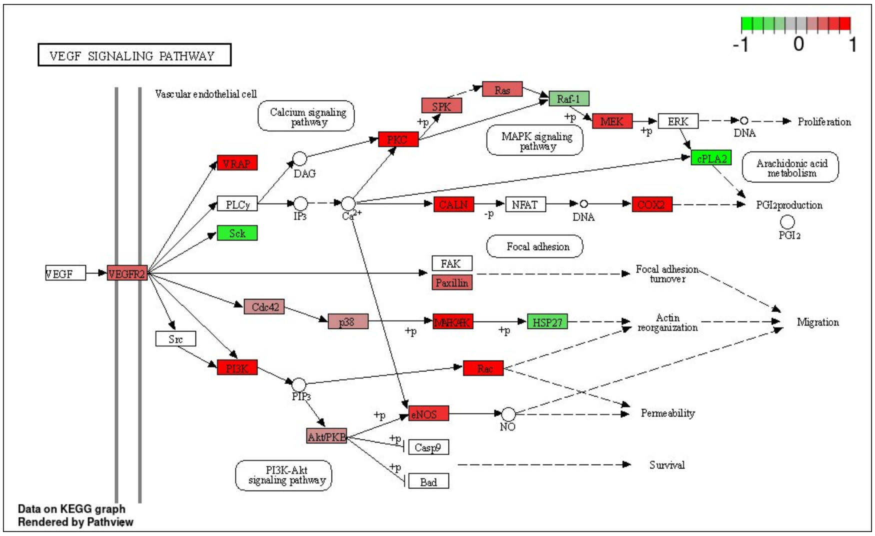

Figure S1B Cell junction pathways

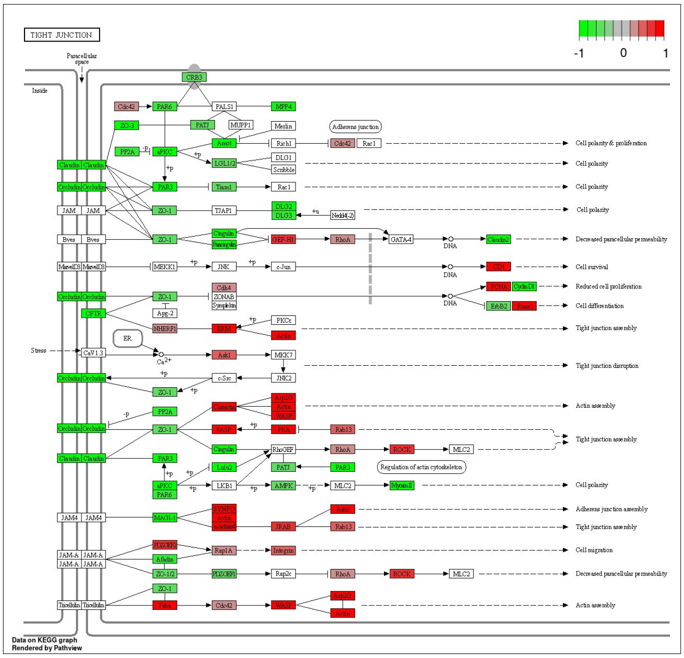

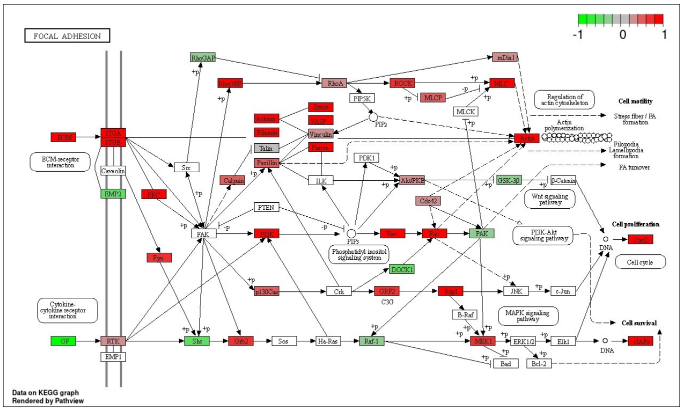

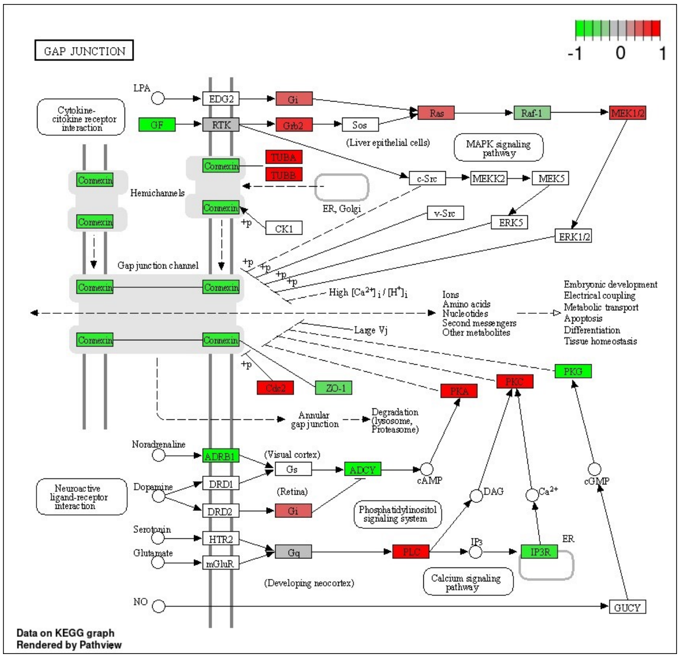

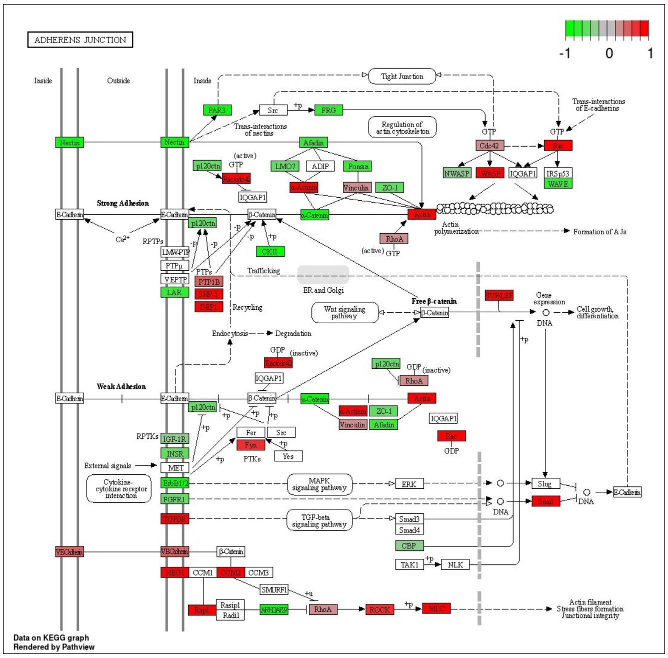

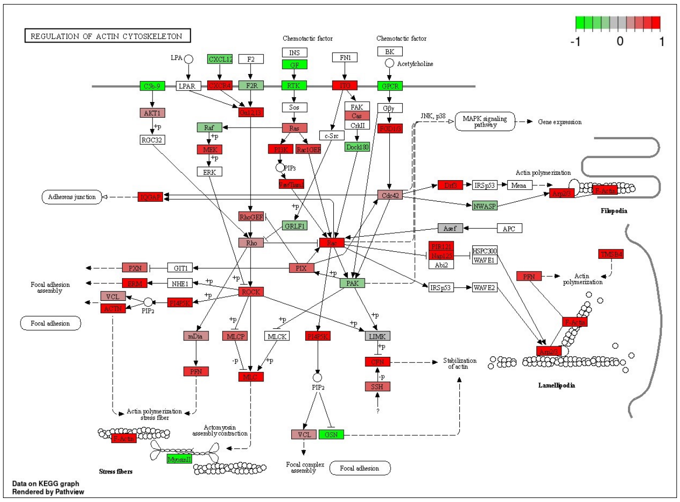

Figure S1C PAMP receptor recognition (PRR) pathways

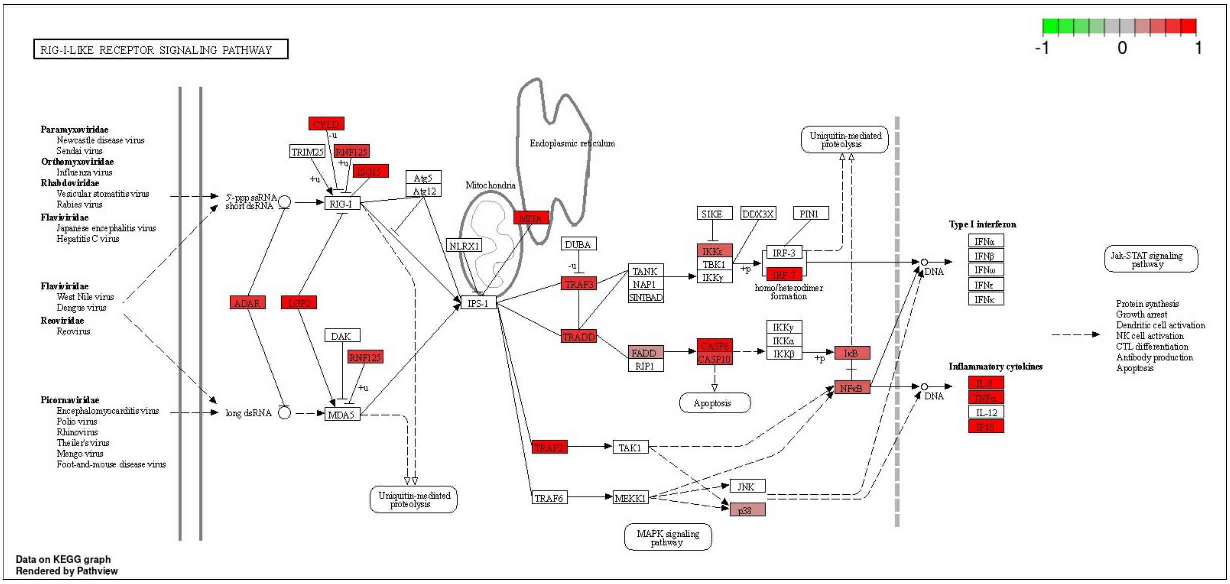

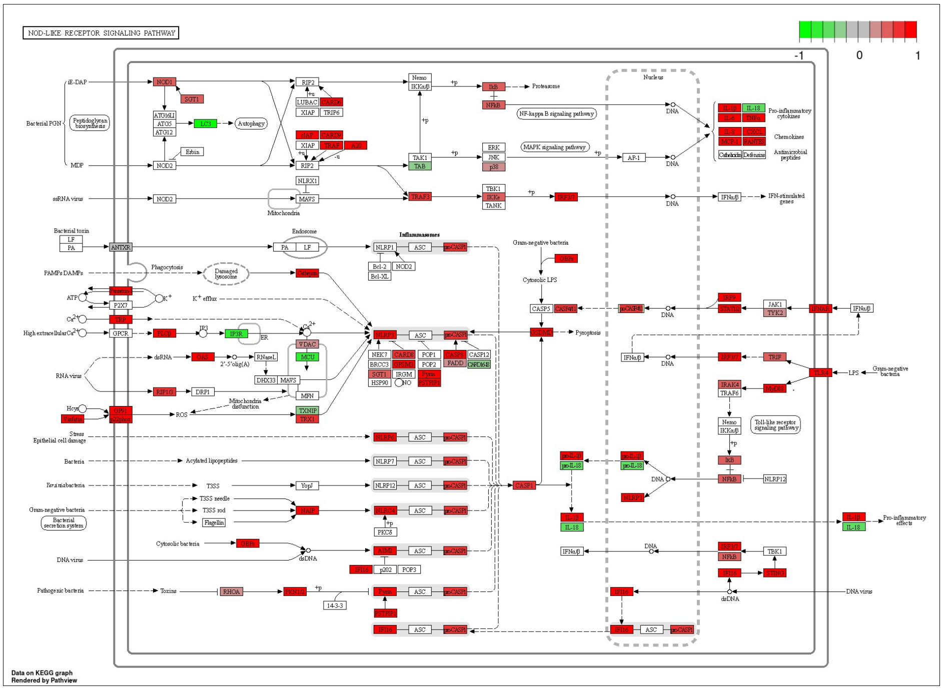

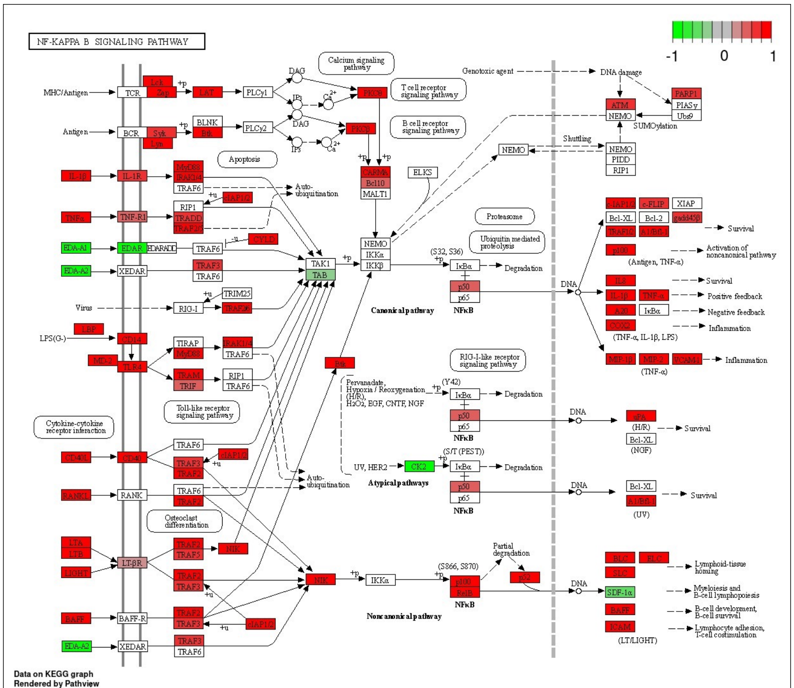

Figure S1C PAMP receptor recognition (PRR) pathways (continued).

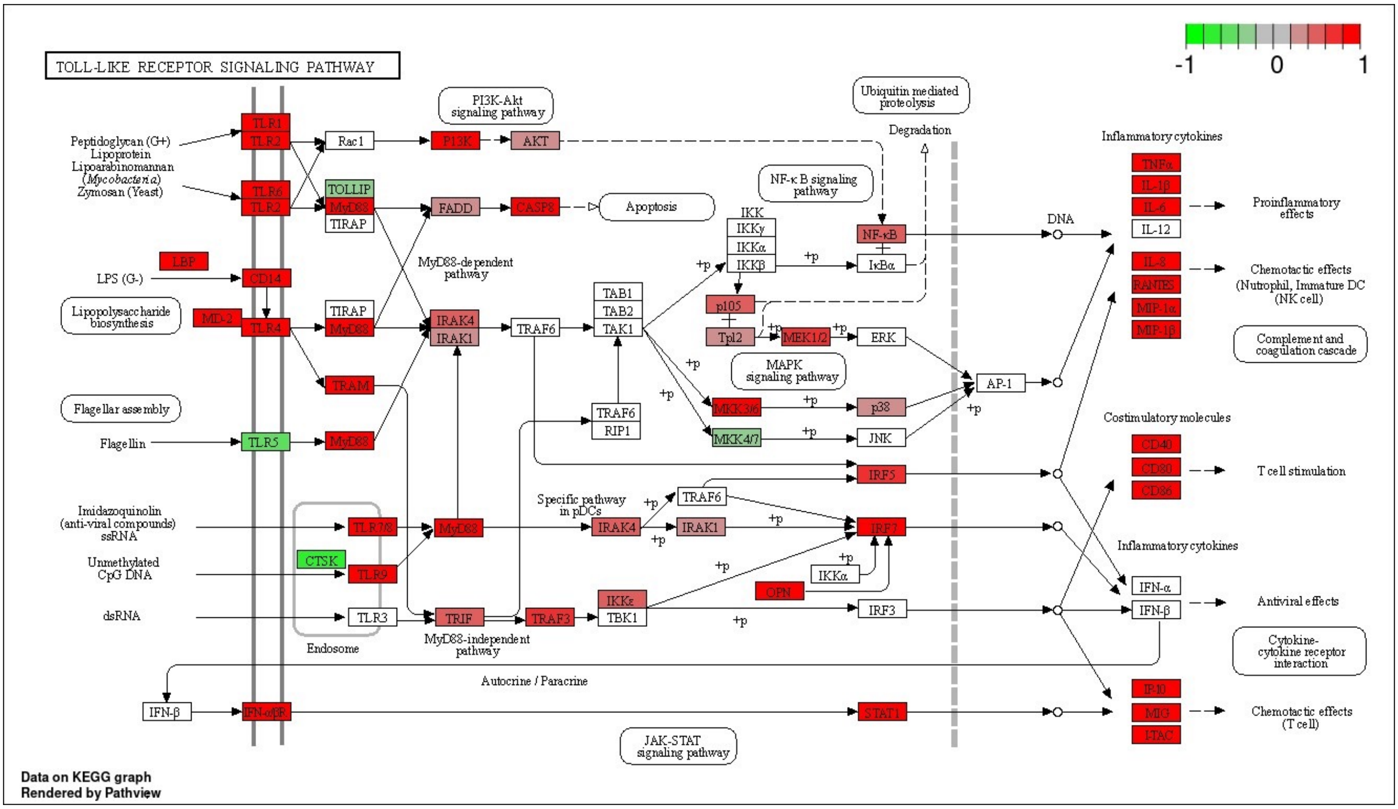

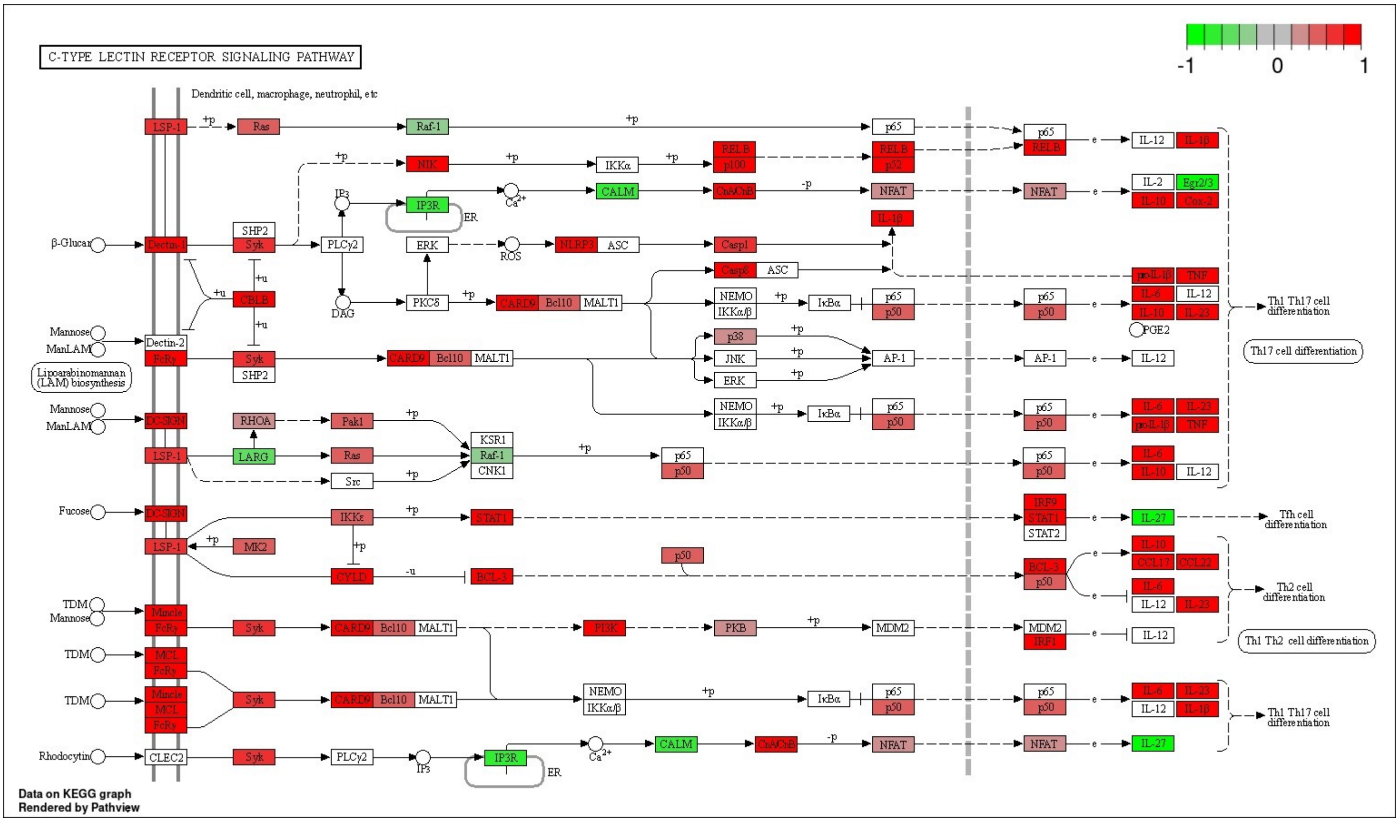

Supplementary Figure 2 – Prime/boost Montanide adjuvanted AGSv-PLUS vaccination modulates immune gene expression response to *Ae.aegypti* mosquito biting.

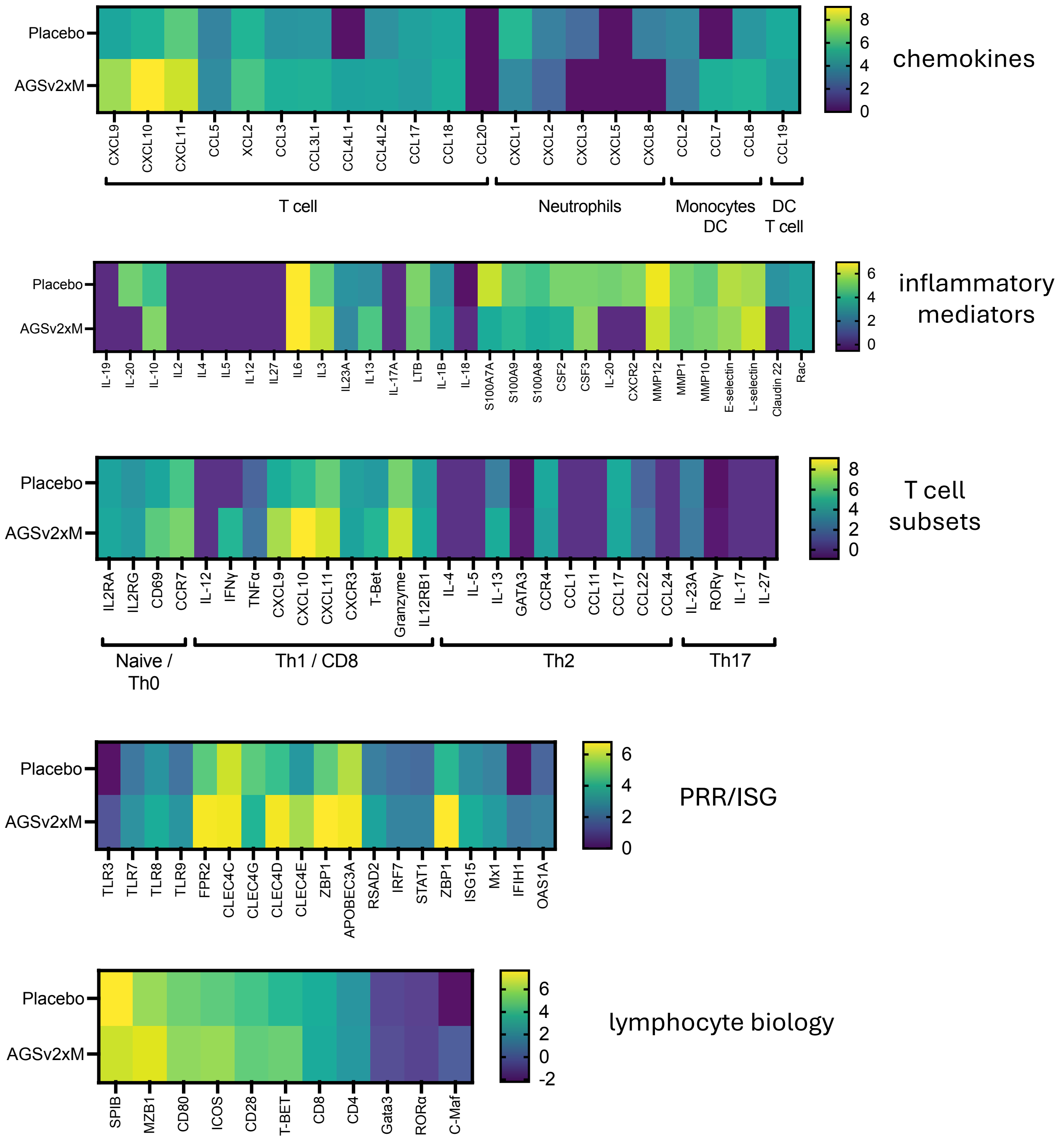

Supplement: Supplementary file 1 [file vaccines-13-01026-s001.zip › supplementary figures.pdf]
