# Supplementary material for: AGS-v PLUS, a Mosquito Salivary Peptide Vaccine, Modulates the Response to Aedes Mosquito Bites in Humans
Source: Vaccines (Basel). 2025 Sep 30;13(10):1026. doi: 10.3390/vaccines13101026 (PMC12567680; doi:10.3390/vaccines13101026)
Supplement: Supplementary file 1 [file vaccines-13-01026-s001.zip › Supplementary Table S1 - gene abbreviations.pdf]

Supplementary Table S1 – abbreviations.

Abbreviation	Full Gene Name
APOBEC3A	Apolipoprotein B mRNA editing enzyme catalytic subunit 3A
C-Maf	Musculoaponeurotic fibrosarcoma oncogene homolog
CCL	C-C motif chemokine ligand
CD	Cluster of differentiation
CLC	Charcot-Leyden crystal protein
CLDN	Claudin
CLEC	C-type lectin domain family
CLEC4E (Mincle)	Macrophage inducible C-type lectin
CSF	Colony-stimulating factor
CXCL	C-X-C motif chemokine ligand
CXCR	C-X-C motif chemokine receptor
FPR2	Formyl peptide receptor 2
GATA3	GATA binding protein 3
GZMB	Granzyme B
ICOS	Inducible T-cell costimulator
IFN	Interferon
IFNGR	Interferon gamma receptor
IL	Interleukin
IRF	Interferon regulatory factor
ISG	Interferon-stimulated gene
JAK	Janus kinase
LILRA5	Leukocyte immunoglobulin-like receptor subfamily A member 5
LTB	Lymphotoxin beta
MAPK	Mitogen-activated protein kinase
MMP	Matrix metallopeptidase
MZB1	Marginal zone B and B1 cell-specific protein
MX1	MX dynamin-like GTPase 1
NF-κβ	Nuclear factor kappa B
NOD	Nucleotide-binding oligomerization domain-containing protein
OAS1A	2'-5'-oligoadenylate synthetase 1A
PYHIN1	Pyrin and HIN domain family member 1
RAC	Ras-related C3 botulinum toxin substrate
RAP1	Ras-proximate-1
RAS	Rat sarcoma viral oncogene homolog

RIG-I	Retinoic acid-inducible gene I
ROR	RAR-related orphan receptor
RSAD2	Radical S-adenosyl methionine domain containing 2
SFRP5	Secreted frizzled-related protein 5
SELE	Selectin E
SELL	Selectin L
SPIB	Spi-B transcription factor
STAT1	Signal transducer and activator of transcription 1
T-BET	T-box expressed in T cells
Th	T helper (subset of T lymphocytes)
TLR	Toll-like receptor
TNF	Tumor necrosis factor
WNT	Wingless-type MMTV integration site family
XCL2	X-C motif chemokine ligand 2
ZBP1	Z-DNA binding protein 1
