# Supplementary material for: AGS-v PLUS, a Mosquito Salivary Peptide Vaccine, Modulates the Response to Aedes Mosquito Bites in Humans
Source: Vaccines (Basel). 2025 Sep 30;13(10):1026. doi: 10.3390/vaccines13101026 (PMC12567680; doi:10.3390/vaccines13101026)
Supplement: Supplementary file 1 [file vaccines-13-01026-s001.zip › Supplementary Table S2- gene ontegny.pdf]

Supplementary Table S2. Gene ontologies of DEG (p>0.01) in *Ae. Aegypti* bitten skin compared to resting skin.

Gene ontologies (P<0.001) / Biological processes / Innate immunity

Innate immunity						
GOBPID gene ontology identifier for biological processes	Pvalue	OddsRatio	ExpCount - expected nymber of observations	Count - actual observations	Number of genes in the array	Term
GO:0002274	0.000257959	6.325931364	7.081582538	15	19	myeloid leukocyte activation
GO:0001774	8.35427E-05	5.991794134	8.646908086	18	23	microglial cell activation
GO:0043011	0.000620991	5.811356297	6.77470196	14	18	myeloid dendritic cell differentiation
GO:1902105	1.83088E-05	4.696755416	12.66425005	25	34	regulation of leukocyte differentiation
GO:0002449	1.01206E-08	3.805745113	29.11376184	54	78	lymphocyte mediated immunity
GO:0032945	2.63583E-08	3.684545318	28.9806695	53	77	negative regulation of mononuclear cell proliferation
GO:0002757	2.79334E-16	3.517437412	67.29704573	121	179	immune response-activating signal transduction
GO:0002768	6.65666E-18	3.264446841	83.93102984	147	223	immune response-regulating cell surface receptor signaling pathway
GO:0002708	5.64874E-07	3.157162418	29.31250422	51	78	positive regulation of lymphocyte mediated immunity
GO:0032943	0.000194601	3.061140804	16.77314657	29	45	mononuclear cell proliferation
GO:0002275	0.000131073	3.042402714	18.03039103	31	48	myeloid cell activation involved in immune response
GO:0002696	1.7E-19	3.037932277	103.6468283	177	276	positive regulation of leukocyte activation
GO:0002285	0.00000269	2.986874404	28.0856039	48	75	lymphocyte activation involved in immune response
GO:0001776	0.0000267	2.800607046	25.19785555	42	67	leukocyte homeostasis
GO:0032946	9.23E-09	2.764607568	48.92840304	81	130	positive regulation of mononuclear cell proliferation
GO:0045639	0.000224455	2.699193291	20.67096643	34	55	positive regulation of myeloid cell differentiation
GO:0002695	1.33E-08	2.658663344	51.47094161	84	137	negative regulation of leukocyte activation
GO:0006954	1.24E-13	2.486964749	100.5740305	160	271	inflammatory response
GO:0002521	2.85E-11	2.399071514	87.3335847	137	235	leukocyte differentiation
GO:0002532	0.000507202	2.342632676	24.45440453	38	65	production of molecular mediator involved in inflammatory response
GO:0045087	9.87E-13	2.278933566	113.4412252	174	306	innate immune response
GO:0030099	0.000000222	2.217609479	63.29290243	96	169	myeloid cell differentiation
GO:0002684	9.28E-10	2.191528272	90.47447699	137	246	positive regulation of immune system process
GO:0050729	0.000419088	2.111913771	32.27832196	48	86	positive regulation of inflammatory response
GO:0002520	7.85E-21	1.955975984	310.6551282	439	827	immune system development

Gene ontologies (P<0.001) / Biological processes / Chemokine activity

Chemokine activity						
GOBPID gene ontology identifier for biological processes	Pvalue	OddsRatio	ExpCount - expected nymber of observations	Count - actual observations	Number of genes in the array	Term
GO:0030595	0.000139363	Inf	3.357484294	9	9	leukocyte chemotaxis
GO:0010818	9.91E-04	14.95635776	3.760366799	9	10	T cell chemotaxis
GO:0070098	0.0000453	2.938523603	21.8003642	37	58	chemokine-mediated signaling pathway
GO:0030593	0.0000262	2.804052832	25.17868952	42	67	neutrophil chemotaxis
GO:0006935	0.000402376	1.70693893	62.31715568	84	168	chemotaxis

Cytokine receptor activity						
GOBPID gene ontology identifier for biological processes	Pvalue	OddsRatio	ExpCount - expected nymber of observations	Count - actual observations	Number of genes in the array	Term
GO:0032695	1.02374E-05	13.29073941	6.77470196	16	18	negative regulation of interleukin-12 production
GO:0032753	5.78615E-06	8.310883621	9.032935947	20	24	positive regulation of interleukin-4 production
GO:0032733	3.36734E-06	4.824986508	14.67852091	29	39	positive regulation of interleukin-10 production
GO:0032703	0.000258383	4.50935023	9.785680609	19	26	negative regulation of interleukin-2 production
GO:0032691	0.000508815	4.271249038	9.409308278	18	25	negative regulation of interleukin-1 beta production
GO:0071353	0.000980688	4.037145424	9.027493261	17	24	cellular response to interleukin-4
GO:0032715	9.96674E-06	3.967636107	16.56038257	31	44	negative regulation of interleukin-6 production
GO:0032743	0.000369159	3.474703912	12.79665926	23	34	positive regulation of interleukin-2 production
GO:0032755	3.64168E-07	3.057985421	31.99164814	55	85	positive regulation of interleukin-6 production
GO:0032757	0.000135734	2.722458737	21.8295952	36	58	positive regulation of interleukin-8 production
GO:0032731	0.000227534	2.646358234	21.45322287	35	57	positive regulation of interleukin-1 beta production
GO:0034097	9.05E-15	2.395898514	119.3353081	187	323	response to cytokine
GO:0019221	0.000717871	2.20054076	26.31098696	40	71	cytokine-mediated signaling pathway

Antigen processing and presentation						
GOBPID gene ontology identifier for biological processes	Pvalue	OddsRatio	ExpCount - expected nymber of observations	Count - actual observations	Number of genes in the array	Term
GO:0002396	0.000310642	9.966265925	5.267448127	12	14	MHC protein complex assembly
GO:0002503	0.000311784	9.960903874	5.269212636	12	14	peptide antigen assembly with MHC class II protein complex
GO:0019885	0.000211689	7.749312283	6.398329629	14	17	antigen processing and presentation of endogenous peptide antigen via MHC class I
GO:0019883	6.23392E-05	7.064756601	7.900821992	17	21	antigen processing and presentation of endogenous antigen
GO:0019886	2.49352E-05	5.817406143	10.16205294	21	27	antigen processing and presentation of exogenous peptide antigen via MHC class II
GO:0019884	2.73721E-06	5.17939794	13.91987871	28	37	antigen processing and presentation of exogenous antigen
GO:0050854	0.000432119	4.721030172	8.639365294	17	23	regulation of antigen receptor-mediated signaling pathway

NFKB						
GOBPID gene ontology identifier for biological processes	Pvalue	OddsRatio	ExpCount - expected nymber of observations	Count - actual observations	Number of genes in the array	Term
GO:1901224	0.0000773	2.663013699	24.46420152	40	65	positive regulation of NIK/NF-kappaB signaling
GO:1901223	0.000610162	3.691730524	10.9147976	20	29	negative regulation of NIK/NF-kappaB signaling
GO:0043123	2.72E-08	2.283587286	68.49976426	105	182	positive regulation of I-kappaB kinase/NF-kappaB signaling
GO:0051092	0.000790143	1.711893929	55.703105	75	148	positive regulation of NF-kappaB transcription factor activity

Cell-cell adhesion						
GOBPID gene ontology identifier for biological processes	Pvalue	OddsRatio	ExpCount - expected nymber of observations	Count - actual observations	Number of genes in the array	Term
GO:0034116	0.000716276	9.129191321	4.892840304	11	13	positive regulation of heterotypic cell-cell adhesion
GO:0022407	1.10903E-17	3.181028419	85.57245141	149	229	regulation of cell-cell adhesion
GO:0007155	0.000000755	1.990054305	74.86268222	109	206	cell adhesion
GO:0034330	0.0000581	1.656285445	92.95069497	123	248	cell junction organization
GO:0030155	3.12571E-05	4.324272219	12.85190926	25	35	regulation of cell adhesion
GO:0045785	6.38143E-23	3.381892949	103.7785253	184	277	positive regulation of cell adhesion

Leukocyte tansendothelial migration						
GOBPID gene ontology identifier for biological processes	Pvalue	OddsRatio	ExpCount - expected nymber of observations	Count - actual observations	Number of genes in the array	Term
GO:0097529	0.00000792	2.741634668	29.59818034	49	79	myeloid leukocyte migration
GO:0007229	0.000161512	2.154939146	35.35768194	53	94	integrin-mediated signaling pathway

interferon responses						
GOBPID gene ontology identifier for biological processes	Pvalue	OddsRatio	ExpCount - expected nymber of observations	Count - actual observations	Number of genes in the array	Term
GO:0035456	0.000399193	Inf	3.008902077	8	8	response to interferon-beta
GO:0034341	7.41968E-06	10.05016358	7.864848361	18	21	response to interferon-gamma
GO:0060337	0.000710113	9.141932002	4.888603835	11	13	type I interferon signaling pathway
GO:0032689	1.3501E-06	5.361675761	14.30214858	29	38	negative regulation of interferon-gamma production
GO:0032729	4.18933E-09	4.39071677	25.96969085	50	69	positive regulation of interferon-gamma production
GO:0071346	0.0000141	2.609544837	30.81708387	50	82	cellular response to interferon-gamma
GO:0034340	0.000620307	2.494239424	20.70047821	33	55	response to type I interferon

Tumour necrosis factor binding						
GOBPID gene ontology identifier for biological processes	Pvalue	OddsRatio	ExpCount - expected nymber of observations	Count - actual observations	Number of genes in the array	Term
GO:0032720	0.0000464	2.995353026	21.07685054	36	56	negative regulation of tumor necrosis factor production
GO:0032760	0.00000487	2.568588838	35.37899912	57	94	positive regulation of tumor necrosis factor production

Vascular effects						
GOBPID gene ontology identifier for biological processes	Pvalue	OddsRatio	ExpCount - expected nymber of observations	Count - actual observations	Number of genes in the array	Term
GO:0051000	0.000761713	4.981518033	7.527446622	15	20	positive regulation of nitric-oxide synthase activity
GO:1901698	0.000000937	1.391992152	346.942322	416	922	response to nitrogen compound

Adaptive immune response						
GOBPID gene ontology identifier for biological processes	Pvalue	OddsRatio	ExpCount - expected nymber of observations	Count - actual observations	Number of genes in the array	Term
GO:0002250	2.11397E-17	5.612118059	41.19548356	85	111	adaptive immune response
GO:0002252	1.76336E-09	4.019503546	29.53094643	56	80	immune effector process
GO:0002683	1.45388E-06	3.804889314	20.42077331	38	55	negative regulation of immune system process
GO:0002822	0.000315656	3.690995261	11.97228637	22	32	regulation of adaptive immune response based on somatic recombination of immune receptors built from immunoglobulin superfamily domains
GO:0002460	1.90637E-07	3.615961125	25.72665848	47	69	adaptive immune response based on somatic recombination of immune receptors built from immunoglobulin superfamily domains
GO:0002699	1.49221E-07	3.30763583	29.92113136	53	80	positive regulation of immune effector process
GO:0002820	0.00013839	2.889212137	19.57049512	33	52	negative regulation of adaptive immune response
GO:0002824	0.0000149	2.744541315	27.80055327	46	74	positive regulation of adaptive immune response based on somatic recombination of immune receptors built from immunoglobulin superfamily domains
GO:0002376	0.000638346	2.657692308	16.60434372	28	48	immune system process
GO:0050776	5.56E-12	2.613702624	77.29818436	126	210	regulation of immune response

T-cell biology						
GOBPID gene ontology identifier for biological processes	Pvalue	OddsRatio	ExpCount - expected nymber of observations	Count - actual observations	Number of genes in the array	Term
GO:0035743	1.10344E-05	23.25296017	5.645584967	14	15	CD4-positive, alpha-beta T cell cytokine production
GO:0050860	2.85795E-05	7.477109515	8.280191284	18	22	negative regulation of T cell receptor signaling pathway
GO:0050870	5.38157E-05	7.177777778	7.823080071	17	21	positive regulation of T cell activation
GO:0042110	6.10604E-05	6.190857575	8.472089314	18	23	T cell activation
GO:0031295	1.34362E-06	5.363410927	14.29928581	29	38	T cell costimulation
GO:0001916	0.000107539	5.261853448	9.408891883	19	25	positive regulation of T cell mediated cytotoxicity
GO:0043029	0.000504318	4.275543227	9.403437816	18	25	T cell homeostasis
GO:0042102	1.8553E-06	3.863516021	19.90632176	37	53	positive regulation of T cell proliferation
GO:0002292	2.43436E-06	3.50877193	22.19609033	40	59	T cell differentiation involved in immune response
GO:0002287	2.84561E-06	3.255930814	24.46420152	43	65	alpha-beta T cell activation involved in immune response
GO:0050852	0.0000144	2.710458665	28.53399959	47	76	T cell receptor signaling pathway

B-cell biology						
GOBPID gene ontology identifier for biological processes	Pvalue	OddsRatio	ExpCount - expected nymber of observations	Count - actual observations	Number of genes in the array	Term
GO:0045577	0.000408241	16.61577151	4.137240356	10	11	regulation of B cell differentiation
GO:0002381	0.000207684	7.768523526	6.388498446	14	17	immunoglobulin production involved in immunoglobulin-mediated immune response
GO:0030889	0.000211689	7.749312283	6.398329629	14	17	negative regulation of B cell proliferation
GO:0016064	0.000975629	6.686989657	5.620801954	12	15	immunoglobulin mediated immune response
GO:0050853	9.53333E-05	3.997260768	12.78124789	24	34	B cell receptor signaling pathway
GO:0030183	0.000689807	2.075150708	31.17962738	46	83	B cell differentiation
